# Predictors of Three Dose On-Time Compliance with HPV4 Vaccination in a Disadvantaged, Underserved, Safety Net Population in the US Midwest

**DOI:** 10.1371/journal.pone.0071295

**Published:** 2013-08-08

**Authors:** Inge Verdenius, Diane M. Harper, George D. Harris, R. Stephen Griffith, Jeffrey Wall, Laura K. Hempstead, Gerard J. Malnar, Ruud L. M. Bekkers

**Affiliations:** 1 Radboud University, Nijmegen, The Netherlands; 2 Center of Excellence, Women’s Health, University of Missouri–Kansas City School of Medicine, Kansas City, Missouri, United States of America; 3 Departments of Biomedical and Health Informatics, University of Missouri–Kansas City School of Medicine, Kansas City, Missouri, United States of America; 4 Department of Obstetrics and Gynecology, University of Missouri–Kansas City School of Medicine, Kansas City, Missouri, United States of America; 5 Department of Community and Family Medicine, University of Missouri–Kansas City School of Medicine, Kansas City, Missouri, United States of America; 6 Department of Obstetrics and Gynecology, Radboud University, Nijmegen, The Netherlands; University of North Carolina School of Medicine, United States of America

## Abstract

**Background:**

HPV4 is approved as a series of three timed doses expected to result in efficacy against specific HPV infections. Completion rates in the US are quite low at the same time the structure of health care delivery is changing. The aim of this study was to determine how the patient-, clinic- and systems-level characteristics facilitate or hinder the timely completion of three HPV4 doses in both adolescent and adult female populations in a high-risk safety net population.

**Methods:**

This is a retrospective study in which patient-, clinic- and systems-level data are abstracted from the electronic medical record (EMR) for all females 10–26 years of age receiving at least one dose of HPV4 between July 1, 2006 and October 1, 2009.

**Results:**

Adults were more likely to complete the three dose series if they had at least one health care visit in addition to their HPV4 visit, (aOR = 1.54 (95% CI:1.10, 2.15). Adults were less likely to complete the three dose series if they received their second HPV4 dose at an acute health care, preventive care or postpartum visits compared to an HPV4-only visit (aOR = 0.31 (95% CI: 0.13, 0.72), 0.12 (0.04, 0.35), 0.30 (0.14, 0.62), respectively). Hispanic adults were less likely than whites to complete the series (aOR = 0.24 (95% CI:0.10, 0.59). 39% of adolescents who completed two doses completed the series.

**Conclusions:**

HPV4 is more likely to be effectively administered to adults in a safety net population if multiple health care needs can be met within the health care system.

## Introduction

Cervical cancer has continued to decline in the US to 8.0/100,000 because of Pap screening programs that depend on colposcopy and excisional treatments [1]. The two prophylactic human papillomavirus (HPV) vaccines, approved to prevent HPV 16 and 18, prevent, to various degrees and for uncertain durations, two (HPV4) and seven (HPV2) oncogenic HPV types associated with cervical cancer [2]–[4]. Both vaccines are efficacious in a three dose regimen for reducing the incidence of cervical intraepithelial neoplasia grade 3 (CIN 3), and the need for colposcopies and excisional procedures [5].

HPV vaccination programs in the US indicate that while 53% of 13–17 year old females have received at least one dose of HPV4, only 37% of these have received three in a timely manner [6]. Independent studies in settings including those with Medicaid coverage, private insurance, closed health care systems and university teaching systems also show low rates of completion [7]–[25]. Three studies report the completion rates in safety net health systems to be 12–28% [12], [17], [24].

Safety-net systems serve all ages of vulnerable, uninsured Americans [26], [27]. They are often publically funded and can be either in urban cores or in rural settings. Examples include Federally Qualified Health Centers (FQHC) whose purpose is to enhance the provision of primary care services in these populations [26]. The National Cancer Institute (NCI) Center to Reduce Cancer Health Disparities (CRCHD) postulates that cervical cancer is an indicator of larger health system concerns, a bellwether for other health care vulnerabilities [27], [28]. Hence, optimizing HPV vaccine strategies in safety net health systems may reduce the risk of cervical cancer and improve other health care disparities [27], [29], [30].

The modified Andersen Behavioral Model [31], [32] consists of patient-, clinic- and systems-level characteristics that may influence the behavior to complete three doses of HPV4 on time. Patient-level characteristics that may predict decision making skills about a vaccine to prevent a sexually transmitted infection include age, race/ethnicity, prior pregnancy history, and prior abnormal cytology screenings. Health seeking and decision making behaviors of pediatric and adolescent females are often dominated by parental influence and differ from health behaviors of independent adult women. However, the effect of pregnancy or prior abnormal cytology may mitigate the influence of age in decision making and health seeking behaviors for HPV vaccination. In addition, the health belief systems of different racial/ethnic cultures influence the health decision making processes for cancer prevention [28].

Clinic-level characteristics may describe the intended purpose of the health care visit. Within the behavioral model the intended purpose of the health care visit will influence decision making at the visit. The preventive health care visits root from a different intention than acute care visits or follow up from acute care; likewise, counseling visits, for any reason, engender a different health intention than do visits linked to the delivery of an infant. Understanding how these factors influence the decision to complete an on-time three dose series may allow safety net health systems to focus on vaccination attempts where they are most likely to be successful with minimal resource wastage.

Influential systems-level characteristics are described by those policies that determine overall clinic function. These include the opportunity for repeated health care utilization, allowing multiple health issues to be discussed during specific types of visits, and a program to promote HPV4 vaccination where a standing order for vaccination was in place to facilitate vaccination without a physician visit.

Guided by this modified Andersen behavioral model of access to health care and health systems utilization [31], [32], we designed this study to investigate the integration of the patient-, clinic-, and systems-level characteristics within our safety-net health system on the timely compliance of the HPV4 series completion among adolescent and adult females prior to the approval of HPV2.

## Results

**Figure 1:**
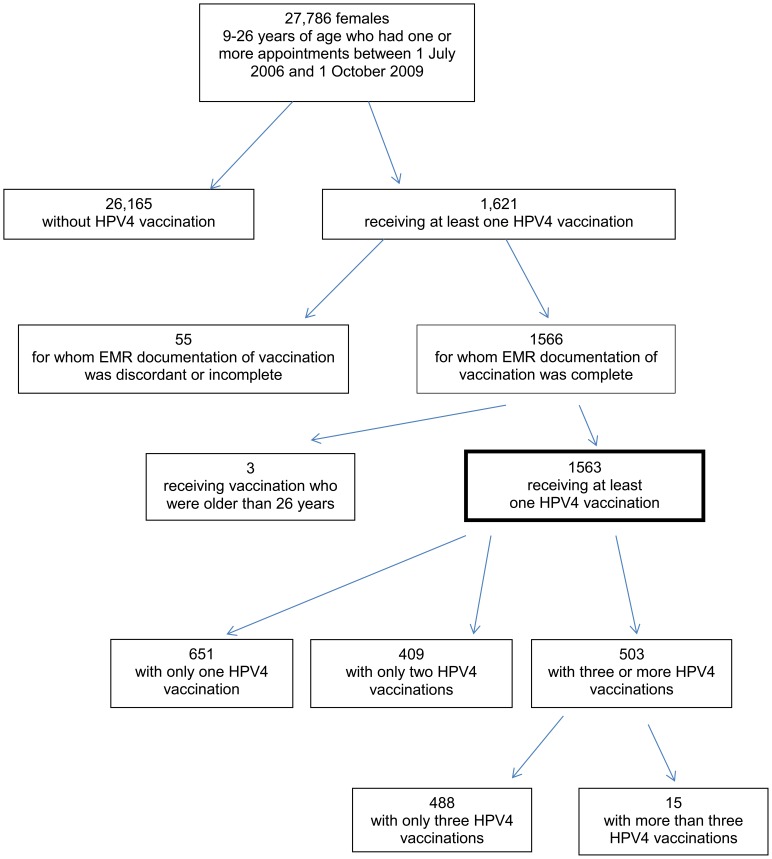
CONSORT diagram of HPV4 recipients in safety net health system.

Of the 27,786 females, aged 10–26 years, 1621 (5.8%) received at least one dose of HPV4 and of those, 1563 had supporting EMR documentation of vaccination (Figure 1). 651 (42%) females received only one dose, 409 (26%) received only two doses, and 503 (32%) received three or more doses of HPV4. Women with more than three doses (0.9%), or who were older than 26 years at the time of their first vaccination were censored from further data analysis.

**Patient-level data** revealed that 258 (17%) were 10–17 year olds (adolescents) and 1305 (83%) were 18–26 year olds (adults) (**Table 1**). Race/ethnicity was reported as 38% Black/50% White in the adolescent group and proportionately reversed in the adult group (57% Black/32% White). Nearly three quarters of all females had at least one pregnancy prior to HPV4 vaccination, including 6% of the 10–13 year olds; and 30% of those screened had past abnormal cytology.

**Table 1:** Descriptors of the Vaccinated Population.

	10–13 year olds	10–17 year olds†	18–26 year olds
	N = 52	N = 258	N = 1305
**Age, mean (SD)**	11.8 (1.0)	15.2 (2.0)	22.0 (2.4)
**Race/ethnicity, n (%)**			
** White**	34 (65%)	129 (50%)	418 (32%)
** Black**	11 (21%)	97 (38%)	739 (57%)
** Hispanic**	3 (6%)	19 (7%)	98 (7%)
** Other**	4 (8%)	13 (5%)	50 (4%)
**Obstetrical History** *		N = 243	N = 1225
** Gravidity, n ≥1 (%)**	3 (6%)	102 (42%)	974 (80%)
** Parity, n ≥1 (%)**	3 (6%)	91 (37%)	927 (76%)
**Obstetrical History** *	N = 52	N = 243	N = 1225
** Gravidity, mean (95% CI)**	0.06 (−0.008, 0.123)	0.52 (0.432, 0.613)	1.64 (1.555, 1.712)
** Parity, mean (95% CI)**	0.06 (−0.008, 0.123)	0.42 (0.346, 0.494)	1.26 (1.202, 1.320)
**Pap cytology** * **n (%)**	N = 0	N = 63	N = 1033
** Abnormal**	–	13 (21%)	317 (31%)

† There were no 9 year olds who received HPV4 in this study. The demographics of the 10–13 year olds were highlighted from the adolescent age group patient-level characteristics because they are a broad CDC target group for HPV vaccination.

* Documented at any time prior to the first HPV4 dose.

**Clinic- and systems-level characteristics** (**Table 2**) showed that the first HPV4 dose was administered most often at a HPV4-only visit for both age groups (52%). Thereafter, 14% of the initiating doses among adolescents occurred equally at either a preventive or postpartum visit; and, for adults, more often at a postpartum visit than a preventive visit (24% vs. 10%, p<0.001). Both concomitant vaccines (45%) and birth control prescriptions (26%) were frequently received at the first HPV4 dose regardless of age.

**Table 2:** Clinic- and Systems-Level Characteristics for Each HPV4 Dose.

	1^st^ Dose			2^nd^ Dose				3^rd^ Dose		
	10–17 yrs	18–26 yrs	All ages	10–17 yrs	18–26 yrs	All ages	10–17 yrs		18–26 yrs	All ages
**Reason for Visit, n (%)**	**N = 258**	**N = 1305**	**N = 1563**	**N = 188**	**N = 724**	**N = 912**	**N = 119**		**N = 384**	**N = 503**
Preventive	36 (14%)	131 (10%)	167 (11%)	10 (5%)	33 (5%)	43 (5%)	17 (14%)		41 (11%)	58 (12%)
Acute Visit	16 (6%)	127 (10%)	143 (9%)	8 (4%)	33 (5%)	41 (5%)	10 (8%)		24 (6%)	34 (7%)
Follow up from an acute visit	7 (3%)	25 (2%)	32 (2%)	4 (2%)	24 (3%)	28 (3%)	4 (3%)		19 (5%)	23 (5%)
Postpartum	36 (14%)	318 (24%)	354 (23%)	10 (5%)	63 (9%)	73 (8%)	3 (3%)		8 (2%)	11 (2%)
Other	14 (5%)	40 (3%)	54 (4%)	6 (3%)	45 (6%)	51 (6%)	7 (6%)		28 (7%)	35 (7%)
HPV4-only	149 (58%)	664 (51%)	813 (52%)	150 (80%)	526 (73%)	676 (74%)	78 (66%)		264 (69%)	342 (68%)
**Selective Prescriptions/Vaccinations Received at Visit** **By Medication Class, n (%)**	**N = 79**	**N = 291**	**N = 370**	**N = 17**	**N = 98**	**N = 115**	**N = 19**		**N = 73**	**N = 92**
Other vaccinations*	37 (47%)	128 (44%)	165 (45%)	10 (59%)	22 (22%)	32 (28%)	6 (32%)		8 (11%)	14 (15%)
Birth Control†	23 (29%)	74 (25%)	97 (26%)	4 (24%)	40 (41%)	44 (38%)	7 (37%)		31 (43%)	38 (41%)
Antibiotics	3 (4%)	33 (11%)	36 (10%)	1 (6%)	18 (18%)	19 (17%)	1 (5%)		15 (21%)	16 (17%)
Antimycotics	1 (1%)	11 (4%)	12 (3%)	0 (0%)	7 (7%)	7 (6%)	2 (11%)		7 (10%)	9 (10%)
**Number of Prescriptions** ‡ **per visit, mean (95% CI)**	0.31 (0.21, 0.412)	0.22 (0.18, 0.26)	0.24 (0.21, 0.27)	0.09 (0.04, 0.14)	0.14 (0.11, 0.17)	0.13 (0.10, 0.16)	0.16 (0.09, 0.23)		0.19 (0.14, 0.24)	0.18 (0.14, 0.22

* Other vaccines given at the same time as HPV4 include TDaP, Td, Hep A, HepA/HepB combination, seasonal flu vaccine, meningitis (MCV4), MMR, pneumococcal vaccine, or varicella virus vaccine.

† Birth control includes oral contraceptive pills, OrthoEvra® patch, Nuvaring®, or Depo-Provera® injection.

‡ Prescriptions include 35 classes of pharmacy dispensed prescriptions in all forms (injectable, oral, suppository, topical, or inhaler) and other vaccines received at the visit, excluding HPV4 vaccination.

CI means confidence intervals.

The second dose was most often received at a HPV4-only visit for both age groups (74%) along with concomitant vaccinations and birth control prescriptions: 59% and 24%, respectively, for adolescents, and 22% and 41%, respectively, for adults.

Less than a third (503/1563) of the females initiating the HPV4 series completed three doses regardless of age. The final dose was received most often at a HPV4-only visit (68%), followed by a preventive health care visit (12%).

### On-time Series Completion of Three Doses

The three dose series was completed, on average, within 30 weeks (95% CI: 29, 31) of initiating the series, regardless of age or number of visits: 53% attended only the three HPV4 visits; 47% attended, on average, 5.4 visits (95% CI: 5.2, 5.6). 18% of the population took longer than one year to complete the three dose series at an average of 83 weeks (95% CI: 80, 86).

Of the females who received three doses (**Table** **3**), 68% (342/503) received them within the recommended time intervals. Of females receiving at least one HPV4 dose, more adolescents than adult women completed the series on time (29% (74/258) vs. 21% (268/1305), p = 0.004), but among those receiving at least two doses, adolescents and adults equally completed the series (39.4% (74/188) vs. 37.0% (268/724)). Predicting receipt of the second dose within the series was only dependent on patient-level characteristics: black adolescents were less likely than white adolescents (OR = 0.27 (95% CI: 0.11, 0.71)); and older adults (22–26 yrs) were more likely than younger adults (18–21 yrs) (OR = 1.80 (95% CI: 1.16, 2.79)).

**Table 3:** Timely Series Completion by Visit Type at Each HPV4 Dose.

	On-Time Three Dose Series Completion*									
	First HPV4 Dose			Second HPV4 Dose				Third HPV4 Dose		
	10–17 yrs	18–26 yrs	All women	10–17 yrs	18–26 yrs	All women	10–17 yrs		18–26 yrs	All women
	N = 74	N = 268	N = 342	N = 74	N = 268	N = 342	N = 74		N = 268	N = 342
Preventive	18	40	58	3	4	7	4		20	24
	(24%)	(15%)	(17%)	(4%)	(1%)	(2%)	(5%)		(7%)	(7%)
Acute visit	6	34	40	3	8	11	7		15	22
	(8%)	(13%)	(12%)	(4%)	(3%)	(3%)	(10%)		(6%)	(6%)
Follow up visits	4	9	13	3	9	12	3		14	17
	(5%)	(3%)	(4%)	(4%)	(3%)	(4%)	(4%)		(5%)	(5%)
Postpartum	6	37	43	1	9	10	0		0	0
	(8%)	(14%)	(13%)	(1%)	(3%)	(3%)	–		–	–
Other visits	5	11	16	1	17	18	3		21	24
	(7%)	(4%)	(5%)	(1%)	(6%)	(5%)	(4%)		(8%)	(7%)
HPV4-only	35	137	172	63	221	284	57		198	255
	(47%)	(51%)	(50%)	(85%)	(82%)	(83%)	(77%)		(74%)	(74%)
*Prescriptions*	*11*	*38*	*49*	*3*	*20*	*23*	*9*		*35*	*44*
	*(15%)*	*(14%)*	*(14%)*	*(4%)*	*(7%)*	*(7%)*	*(12%)*		*(13%)*	*(13%)*
*Other Vaccinations*	*11*	*6*	*17*	*3*	*3*	*6*	*1*		*3*	*4*
	*(15%)*	*(2%)*	*(5%)*	*(4%)*	*(1%)*	*(2%)*	*(1%)*		*(1%)*	*(1%)*

* Dosing intervals: dose 1–2≥4 weeks but ≤26 weeks; dose 2–3≥12 weeks; and dose 1–3≥24 weeks but ≤52 weeks.

Completion of three on-time doses among those receiving at least one dose occurred at equal frequencies within the dichotomized adolescent and adult age groups. For adolescents there was no difference in on-time completion rates between the younger 10–13 year olds and the older 14–17 year olds (36.5% (19/52) vs. 26.7% (55/206)); and for adults, there was no difference in on-time completion rates between younger 18–21 year olds and older 22–26 year olds (20.1% (114/568) vs. 20.9% (154/737)).

The visit type at which HPV4 was received was dominated by HPV4-only visits among on-time completers. Subsequently, the first HPV4 dose was received at preventive visits in 24% of adolescents and 15% of adults. The third on-time HPV4 dose was received at an acute visit in 10% of adolescents. In adults, the third on-time HPV4 dose was received equally among all visit types, except the postpartum visit. No person completed the third dose on-time at a postpartum visit.

For on-time completers, the frequency of concomitant vaccinations was significantly higher among adolescents (15%) than adults (2%) with the first HPV4 dose (p<0.001); and was significantly higher at the first than at any other HPV4 dose (p<0.001). Prescriptions were provided equally to adolescents (15%) and adults (14%); and for both ages combined, equally at the initiating (14%) and completing doses (13%). Very few prescriptions or concomitant vaccinations were provided at the second HPV4 dose.

Among all females who received at least two doses, age did not predict on-time series completion (**Table 4**). Other patient-, clinic- and systems-level characteristics in the adolescent age group influenced the likelihood of completing three HPV4 doses on-time when considered singly, but, in the adjusted analysis no model-based characteristics predicted adolescent on-time HPV4 completion.

**Table 4:** Predictors of On Time Series Completion by Patient-, Clinic- and Systems-Level Characteristics.

	Receipt of On-Time Three Dose Series*								
	Crude OR (95% CI)		Adjusted§ OR (95%CI)						
	10–17 yrs	18–26 yrs	10–17 yrs	18–26 yrs					
	N = 188	N = 724	N = 180	N = 683					
**Age (yrs)** †	0.93 (0.81, 1.07)	1.02 (0.96, 1.09)	§§	§§					
**Race/ethnicity** †									
White	Referent	Referent	Referent	Referent					
Black	**0.39 (0.20, 0.74)**	0.87 (0.62, 1.21)	0.62 (0.28, 1.33)	§§					
Hispanic	0.30 (0.08, 1.18)	**0.19 (0.08, 0.45)**	§§	**0.24 (0.10, 0.59)**					
Other	**0.08 (0.01, 0.60)**	1.37 (0.66, 2.85)	**0.05 (0.01, 0.48)**	§§					
**Gravidity** †	N = 180	N = 683	N = 180	N = 683					
G = 0	Referent	Referent	Referent	Referent					
G>0	**0.46 (0.24, 0.88)**	**0.45 (0.32, 0.64)**	0.32 (0.04, 3.01)	1.41 (0.58, 3.46)					
**Parity** †	N = 180	N = 683	N = 180	N = 683					
P = 0	Referent	Referent	Referent	Referent					
P>0	**0.52 (0.27, 1.00)**	**0.41 (0.29, 0.57)**	2.18 (0.24, 20.17)	**0.34 (0.15, 0.81)**					
**Visit Type for dose 1 visit**									
HPV4-only	Referent	Referent	Referent	Referent					
Preventive	**3.27 (1.40, 7.68)**	1.34 (0.84, 2.11)	2.11 (0.79, 5.68)	§§					
Acute Visit	1.50 (0.48, 4.66)	1.61 (0.97, 2.68)	§§	§§					
Follow up	4.00 (0.70, 22.91)	1.80 (0.70, 4.65)	§§	§§					
Postpartum	0.67 (0.24, 1.83)	0.66 (0.43, 1.02)	§§	§§					
Other Health Needs	2.00 (0.54, 7.37)	**2.83 (1.07, 7.48)**	§§	1.46 (0.50, 4.25)					
**Visit Type for dose 2 visit**									
HPV4-only	Referent	Referent	Referent	Referent					
Preventive	0.59 (0.15, 2.38)	**0.19 (0.07, 0.55)**	§§	**0.12 (0.04, 0.35)**					
Acute Visit	0.83 (0.19, 3.60)	**0.44 (0.20, 1.00)**	§§	**0.31 (0.13, 0.72)**					
Follow up	4.14 (0.42, 4.76)	0.83 (0.36, 1.93)	§§	§§					
Postpartum	0.15 (0.02, 1.24)	**0.23 (0.11, 0.48)**	§§	**0.30 (0.14, 0.62)**					
Other Health Needs	0.28 (0.03, 2.42)	0.84 (0.45, 1.57)	§§	§§					
**Non-HPV4 visits between first and last dose**									
None	Referent	Referent	Referent	Referent					
≥1 non-HPV4 visit	0.65 (0.35, 1.23)	**1.61 (1.19, 2.18)**	§§	**1.54 (1.10, 2.15)**					
**Other vaccinations at dose 1 visit**									
None	Referent	Referent	Referent	Referent					
≥1 other vaccination type	**3.14 (1.10, 8.91)**	0.67 (0.26, 1.76)	2.66 (0.75, 9.42)	§§					
**Other vaccinations at dose 2 visit**									
None	Referent	Referent	Referent	Referent					
≥1 other vaccination type	0.92 (0.21, 3.98)	0.29 (0.09, 1.01)	§§	§§					
**Other Prescriptions at dose 1 visit**									
None	Referent	Referent	Referent	Referent					
≥1 other prescription	1.82 (0.73, 4.52)	**1.93 (1.19, 3.13)**	§§	1.54 (0.87, 2.71)					
**Other Prescriptions at dose 2 visit**									
None	Referent	Referent	Referent	Referent					
≥1 other prescription	2.37 (0.39, 14.51)	0.74 (0.43, 1.28)	§§	§§					

* Among women with two or more doses and with complete data. Appropriate dosing interval means: the interval between dose 1 and dose 2≥4 weeks but ≤26 weeks, and the interval between dose 2 and dose 3≥12 weeks, and the interval between dose 1 and dose 3≥24 weeks but ≤52 weeks.

§ Adjusted for significant variables in the univariate model.

§§ Variable was not significant in the univariate model.

† Visit at which the first dose of HPV4 was initiated.

Bold font indicates Odds Ratios are statistically significant compared to referent.

In adult women, the adjusted analysis for on-time HPV4 completion was influenced by the model-based characteristics. Specifically, the receipt of other health care between HPV4 doses and the opportunity to receive HPV4 without a physician visit were significant predictors of on-time completion. Additionally, white women were more likely than Hispanics, and nulliparous women were more likely than multiparous women to complete the three dose series on-time.

## Discussion

This study was conducted in a safety net health system with patients at high risk for adverse health outcomes at earlier ages, including cervical cancer [27], [28], as evidenced by 30% of the population already having an abnormal Pap test prior to vaccination. A conceptual framework was used to identify patient-, clinic- and systems-level factors’ influence on behavior to complete the vaccine series. As seen in other underserved populations [12], [17], [24], our rate of on-time adolescent and adult three-dose completion is lower than national data [10], [33].

Our results are the first to show the association between the systems-level policies and on-time HPV4 series completion. Several studies have indicated that a regular source of health care, even among federally qualified health centers (FQHCs) which resemble our safety-net system [29], [30], results in better health prevention outcomes. In addition our results are the first to show among those receiving care in a safety-net population that standing order policies which facilitated receipt of the second HPV4 dose without a physician visit were successful.

In both the adolescent and adult populations our first HPV4 doses were administered much more frequently at HPV4-only visits than reported in a university based health care system, but the second and third doses were administered at HPV4-only visits at similar frequencies [18], [19]. Our results differ from others, though, in that the HPV4-only visit type, especially at the second HPV4 dose, was significantly influential in facilitating our adults to complete the series on-time; however, it had no significant influence on adolescent vaccine completion [18].

Our adolescent results showed a higher association between receiving other concomitant vaccines and the first HPV4 dose versus the second or third HPV4 dose, as shown by Dempsey [18]. Nonetheless when our results were adjusted for patient-, clinic-and systems-level characteristics, concomitant vaccinations did not significantly influence our on-time adolescent completion of the series. This is a disappointment, especially for the high risk safety net population, as the new adolescent platform of vaccines recently recommended by the Advisory Committee on Immunization Practices (ACIP) was meant to reinforce adolescent health care [34], [35].

Patient-level characteristics variously studied by others to predict three dose completion rates are parity, race, and age [8]–[25]. While HPV vaccination is recommended prior to the onset of sexual activity because sexual activity is the most common mechanism of HPV infection [5], virginity has not been shown to be a predictor of completing a three dose HPV4 series. Parity, though, is a strong predictor of not completing a three dose series among 18–26 year old women [13] as our data corroborated.

The influence of race is unclear in the literature, and probably not significant given the wide range of evidence for racial influence on three dose HPV4 completion. Among adolescents in populations similar to our safety net population, Perkins [17] showed that race was not a predictor when considering white, black and Hispanic races; but Cook’s [12] results indicated that black adolescents were less likely than white adolescents to complete the HPV4 series. In other insured populations, white adolescents were more likely to complete the HPV4 series than black or Hispanic adolescents [9], [11], [13]–[15], [18], [20], [21], [23]. Our results, in our safety net population, indicated that white, black and Hispanic adolescents were equally likely to complete three HPV4 doses on-time.

No studies in safety net populations have documented the influence of race on adult completers of the HPV4 series. In various insured adult women populations, race was neither predictive of three dose completion [13] nor more likely for white adults than black or Hispanic adults [14], [15], [19], [23]. Our results indicate that Hispanic women in a safety net population are less likely to complete the three HPV4 doses on-time than white women, but this may be due to the imbalanced racial outreach in our health care system and not intrinsic to Hispanic women.

The age at which to initiate HPV4 has been vociferously debated in the US. Based on the efficacy and immunogenicity data submitted to the FDA for regulatory approval, HPV4 has minimal efficacy if given to those already seropositive for the vaccine-relevant HPV types [36] and inferior antibody titer induction when given in less than three doses [37]–[39]. The CDC recommendation of targeting 11–12 year olds for vaccination assumes this is the optimal age range for HPV vaccine efficacy due to HPV naivety. But even at this young age, HPV naivety is not guaranteed. Up to 7% of females report having sex before age 13, 8% of 9^th^ grade US females (14–15 years) report being forced to have sex, and on average 29% of 9^th^ graders have ever had sex [40]. Other CDC studies show that 10–15% of children from birth through 13 years of age test positive for oro-and anogenital high-risk HPV type infections [5], [41], [42]. Moving the age of vaccination younger or even to infancy risks waning of vaccine efficacy prior to the age range at which HPV prevalence is the highest, 16–25 years [43].

From the opposite perspective, another prevention strategy to ensure effectiveness during the time of highest exposure is to move HPV4 initiation to older adolescents or young adult females; this constitutes the ACIP ‘catch-up’ immunization recommendation. Immunizing at 15 years instead of 12 provides greater cervical cancer prevention for the first 50 years of vaccination assuming that 70% of 15 year olds complete the three doses on-time [44]. This is the most common practice pattern to date in the US [45] despite knowing that by 18 years of age, 64% of females have had a sexual experience, and 23% have had sex with four or more partners [40].

Determining the age of HPV4 initiation, thus, becomes a cost effectiveness question. While no health economic models have directly used compliance with three doses as a modeling parameter, population coverage could be seen as a proxy for dosing compliance. For example, should 70% of the naïve population receive all three doses appropriately, 70% population coverage would be established. If only 50% of this covered population actually completed all three doses on-time, the effective population coverage would drop as low as 35%. Health care models show that the cost effectiveness of HPV4 vaccination wanes dramatically as population coverage diminishes [46]. Hence, timely dosing of the series becomes as important to the health care economics of this primary prevention program as the pivotal parameters of duration of vaccine efficacy and for how many HPV types the female was seronegative at the time of vaccine initiation [47], [48]. If one age range results in significantly inferior three dose completion rates, then the cost-effectiveness of this primary prevention strategy could be compromised.

Nelson’s work [49] shows that for the Hepatitis B vaccine, a similar three dose series, the likelihood of completing the three doses decreases as the age of initiation advances beyond 5 years. For HPV4, several studies show that among adolescents, those younger than 15 years have higher completion rates than older adolescents [11], [12], while other studies show older adolescents are more likely to appropriately complete the HPV4 series than the pre-pubescents ([8]–[10], [23]. Still others show that there is no difference in completion rates by adolescent age of HPV4 initiation [16]–[18], [20], [22], [24]. Our study showed that in a safety net population there was no difference in completion rates between younger and older adolescents.

The same diffuse pattern is seen for adolescent vs. adult completion rates with adolescents more likely to complete the series in some studies [10], [15], [16]; adults more likely to complete the series in other studies [13], [14], [23] and some studies showing that neither age group is more likely than the other to complete the series [17], [19], [21]. Our study showed that in a safety net population, adolescents were more likely than adults to complete the second dose in the series leading to a higher overall completion rate, with white race being the only predictor of second dose completion among adolescents.

Within adult age ranges, while our study supported the literature [19] that age did not predict three dose completion rates, we did show that 22–26 year old women were more likely than 18–21 year old women to complete the second dose in the series. Completing the third dose critically depended on the clinic- and systems-level events at the second dose, leading us to conclude that health care access for all medical needs within a safety net population is more important for complete on-time three dose HPV4 vaccination than the age at which the series is initiated.

### Limitations of the Study

There are several limitations to our study. Our safety net health care system may not be generalizable to other safety net systems, nor to other health care systems with a broad payor mix.

The initiation rate of HPV4 was very low in our institution; hence, those females that did choose to initiate may not be generalizable to the entire safety net population in enthusiasm for completing the series on time. The adolescents served by the TMC health care system may not be generalizable to other pediatric populations in that they are more representative of the safety net population of the uninsured, vulnerable and disenfranchised.

In addition, the on-time series completion rates may currently be worse than this study indicates. This study described the use of HPV4 at a time of high use, immediately after its approval and during the heavy public advertisement campaigns that ensued. In addition, even though it is unlikely given the catchment area and safety net nature of our population, females may have received other HPV4 doses at other health care facilities that we were unable to capture.

## Methods

This research was approved by the TMC Privacy Board and by the UMKC Adult Health Sciences Institutional Review Board as an exempt study not requiring individual consent (#11–16e).

The electronic medical record (EMR) and billing records of Truman Medical Center (TMC) identified all office visits of females whose ages were between 9 and 26 years and who were seen between July 1, 2006 and October 1, 2009. No 9 year olds received HPV4, making our study age range from 10–26 years.

The TMC population is a safety net population [26], [50] of vulnerable uninsured, underinsured and low income patients who seek care in the health care system associated with the teaching service of the University of Missouri-Kansas City School of Medicine; this system is located both in the urban core and in a rural setting at the outer Eastern border of Kansas City, Missouri. Among this set of women, all visits coded for HPV4 vaccination were identified and cross referenced with the vaccine log maintained in the clinic.

Functionally, all data were manually abstracted from the EMR. Patient-level data included age at first HPV4 dose which was recorded as a continuous variable and dichotomized to 10–17 year and 18–26 year groups, mirroring the ages for adolescent and adult abilities to make health care decisions as well as the recognized age of consent for medical care. Race/ethnicity was self-identified in the registration files of the EMR, and coded as White, Black, Hispanic or Other. Gravidity and parity were defined at the first HPV4 dose. Any cytology screening history was recorded through the first HPV4 dose.

Clinic-level data included the date of visit and visit type. The visit type was inferred from the visit note by agreement of two authors, and classified as preventive, acute or follow up from an acute visit, as well as postpartum or any ‘other’ health care visit. Preventive well woman or well child exams included school, sports or work physicals and visits without symptomatic complaints. An acute health care visit was defined as a visit for which a specific health outcome would be addressed such as a new onset illness, a colposcopy or loop electrosurgical excision procedure (LEEP). Follow up for acute health care visits documented the health services rendered subsequent to an acute visit including women in follow up for abnormal cytology. Postpartum visits included those women who received a dose of HPV4 at any time after delivery up to and including the 6 week visit. Other health care visits included counseling visits (e.g. smoking cessation, depression, or contraceptive counseling).

The systems-level visit data included health care visits for more than HPV4 vaccination; the administration of HPV4 without a concomitant physician visit (HPV4-only visit type); and whether other vaccinations or prescriptions were provided at the visit. Other vaccinations given included TDaP (tetanus, diphtheria and acellular pertussis), Td (tetanus and diphtheria), Hepatitis A, Hepatitis A and Hepatitis B combination, seasonal flu vaccine, meningitis (MCV4) vaccine, MMR (measles, mumps and rubella), or varicella virus vaccine. Other prescriptions included topical, oral, vaginal or rectal medications. These characteristics were recorded starting with the first dose of HPV4 and included each health care visit until the last dose of HPV4 was given or 12 months elapsed.

Appropriate time intervals between doses were defined as the interval between dose 1 and dose 2≥4 weeks but ≤26 weeks; the interval between dose 2 and dose 3≥12 weeks; and, the interval between dose 1 and dose 3≥24 weeks but ≤52 weeks as defined by the Centers for Disease Control and Prevention (CDC) and comparative dose schedules for optimal immunologic response [37]–[39], [51], [52], [53].

### Statistics

Chi-square and Student’s t tests were 2 sided using an alpha of 0.05 with Bonferroni corrections for multiple comparisons. A backward stepwise logistic regression was used to determine which patient-, clinic- and systems-level variables were significant for the outcome using p<0.10 for entry and p<0.05 for significance. All data analyses were performed using SPSS version 18.0 [54].

### Conclusions

Completion of three on-time doses of HPV4 is most influenced by health care access for all medical needs, and is independent of a woman’s age and independent of the type of visit at which she is offered the initial vaccination. Improving the cost effectiveness of cervical cancer prevention may include emphasizing to HPV4 recipients the importance of completing all three doses on time.
